# The Pain Medicine Curriculum Framework-structured integration of pain medicine education into the medical curriculum

**DOI:** 10.3389/fpain.2022.1057114

**Published:** 2023-01-09

**Authors:** Elspeth Shipton, Carole Steketee, Eric Visser

**Affiliations:** ^1^ School of Medicine, University of Notre Dame Australia, Fremantle, WA, Australia; ^2^ Curtin Medical School, Curtin University, Perth, WA, Australia

**Keywords:** pain, pain medicine education, curriculum, medical school, framework

## Abstract

Medical practitioners play an essential role in preventing pain, conducting comprehensive pain assessments, as well as promoting evidence-based practices. There is a need for the development of innovative, interprofessional and integrated pain medicine curricula for medical students. The Pain Medicine Curriculum Framework (PMCF) was developed to conceptualise a purposeful approach to the complex process of curriculum change and to prioritise the actions needed to address the gaps in pain medicine education. The PMCF comprises four dimensions: (1) future healthcare practice needs; (2) competencies and capabilities required of graduates; (3) teaching, learning and assessment methods; and (4) institutional parameters. Curricula need to meet the requirements of registration and accreditation bodies, but also equip graduates to serve in their particular local health system while maintaining the fundamental standards and values of these institutions. The curriculum needs to connect knowledge with experience and practice to be responsive to the changing needs of the increasingly complex health system yet adaptable to patients with pain in the local context. Appropriate learning, teaching and assessment strategies are necessary to ensure that medical practitioners of the future develop the required knowledge, skills and attitudes to treat the diverse needs of patients' experiencing pain. The historical, political, social and organisational values of the educational institution will have a significant impact on curriculum design. A more formalised approach to the development and delivery of a comprehensive pain medicine curriculum is necessary to ensure that medical students are adequately prepared for their future workplace responsibilities.

## 1. Introduction

Every medical practitioner has a responsibility to provide care for patients with pain, because management of pain transcends the speciality and clinical setting (1). Medical practitioners play an essential role in preventing pain, conducting comprehensive pain assessments, and promoting evidence-based practices. Treatment of pain is complex and requires consideration of the type of pain, patient comorbidities, patient risk factors for side effects or addiction, and the psychosocial characteristics of the patient experiencing pain (2, 3). Evidence points to a major gap between the increasingly sophisticated knowledge of pain and the prevailing inadequacy of its treatment (4, 5). Obstacles associated with the implementation of evidence-based pain management strategies are complex, and medical curricula design issues have been highlighted as one of the greatest barriers to effective treatment of pain (6–9).

Research has shown that there is a wide variation in the delivery of pain medicine education at medical schools across Australia and New Zealand (10). In general, medical schools in these countries lack well documented and comprehensive pain curricula (10). Indeed, pain medicine content is lacking in medical curriculae internationally (11).

There have been repeated calls for the development of innovative, interprofessional and integrated pain medicine curricula, education and resources by internationally recognized experts in clinical pain medicine and pain education to ensure that medical practitioners entering the workforce are able to deliver safe and effective pain management (12–17). Seven studies have described the process of developing a pain curriculum at individual medical schools in Canada and the USA, and provided details of the teaching and learning activity associated with the course (18–24).

The enormous difficulties involved with introducing a new curriculum cannot be underestimated (25). Universities are under pressure to change in a variety of ways, for instance, curriculum reform has been implemented to address the disparity in health status between Indigenous and non-Indigenous people in Australia and New Zealand (26, 27). Medical schools are complex educational systems that face their own unique cultural and organizational challenges when it comes to transforming curricula. Change is difficult due to long-standing biases towards basic sciences and tertiary care, perceived need to maintain the status quo and territorial protection of power and status (28–31). The medical specialists who are planning the curriculum and teaching the students are often leaders in their field in the clinical healthcare system, and may not perceive a need for transformative change (32). The medical curriculum is under pressure in terms of appropriate content in general due to an ever-increasing body of medical knowledge to be covered in the curricula. Adding pain education to a full medical curriculum of fixed length may not be well received when this necessitates other content is dropped (18).

There is a need for the development of recommendations to enable effective integration of pain medicine education into medical curricula on an international scale (33). This review article will focus on essential components that need to be considered when considering new ways to include pain management in medical curricula, with particular reference to the Australian and New Zealand context.

Theoretical frameworks of curriculum structure and context are useful to assist in articulating and addressing the complexities of curriculum design and development (34). The Four-Dimensional Curriculum Development Framework (4DF) developed by Lee, Steketee, Rogers and Moran provides a template to comprehensively examine the complex and dynamic nature of the pain curricula for medical students (34). It is a useful tool for identifying curriculum priorities and “connecting content and activity with purpose and consequence” (34). It was designed in Australia to generate curriculum and pedagogical discussions crucial to supporting interprofessional education (IPE) as a core component of health professional education curricula (35). The 4DF has proved to be an effective tool used by individuals and institutions for review and development of interprofessional curricula and curriculum redesign (35, 36). The 4DF framework comprises four dimensions: (1) future healthcare practice needs; (2) competencies and capabilities required of graduates; (3) teaching, learning and assessment methods; and (4) institutional parameters.

**Figure 1:**
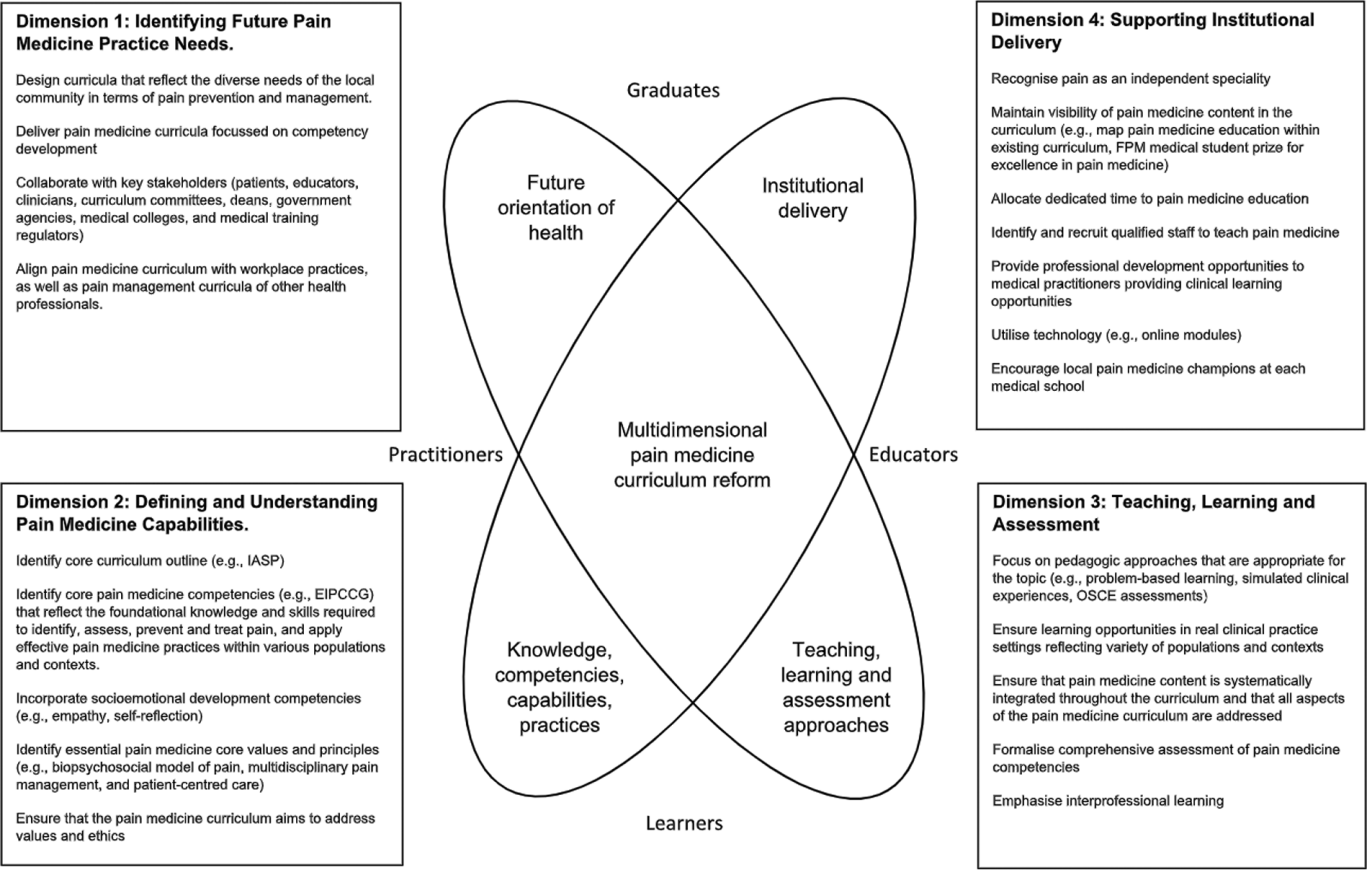
The pain medicine curriculum framework.

The Pain Medicine Curriculum Framework was developed from the 4DF to conceptualise a purposeful approach to the complex process of curriculum change and to prioritise the actions needed to address the gaps in pain medicine education (see Figure 1). This Pain Medicine Curriculum Framework encompasses the four elements of the 4DF with particular reference to the design and delivery of pain medicine education at medical schools (34). PubMed, Medline, EMBASE, ERIC, and Google Scholar, and BEME data bases were searched for information relevant to the four dimensions. The team consisted of four members, two with a medical education lens and two with clinical pain medicine focus. One author, CS, was instrumental in the design of the 4DF and the subsequent application of the 4DF to specific curricula parameters. This was valuable when applying the 4DF to the unique demands of pain education for medical students. While this framework is particularly relevant to the Australian and New Zealand context, the framework is applicable to medical schools internationally with similar medical education systems, when locally contextualised.

## 2. Dimension 1: identifying future healthcare practice needs in pain medicine

The first dimension of this framework asks the questions “What is this curriculum for?” and “What is the professional landscape that it aims to prepare students for, now and in the future?” (35) Curricula need to meet the requirements of registration and accreditation bodies, but also equip graduates to serve in their particular local health system while maintaining the fundamental standards and values of these institutions (34). Curriculum design influences the education of future health professionals in terms of personal, professional, social, cultural, political and economic development, by setting the pre-conditions for the development of specific knowledge, skills and attitudes (34).

### 2.1. Community need for pain medicine education

Why is it important that pain medicine is included in the medical curriculum? Acute pain is an almost universal experience and arises from trauma, burns, infection, emergency and elective surgery, childbirth and severe medical illness. There is a high prevalence of chronic pain in Australia and New Zealand; evidence from large-scale studies show that approximately one in five adults experience chronic pain (37–39). Half of all cancer patients experience chronic pain, and one third will describe their pain as moderate or severe (40). The Global Burden of Disease Study 2016 placed low back pain, migraine, other musculoskeletal pain (such as autoimmune, inflammatory, joint, ligament, tendon and muscle disorders) and neck pain in the top six causes of years lived with disability in Australia and New Zealand, alongside depression and anxiety (41, 42). In 2018, 3.24 million Australians were living with chronic pain, and it was estimated that in 2016–2017, about 770,000 adults in New Zealand experienced pain almost every day (43, 44). Chronic pain is common in children and adolescents, and in the elderly (45–47). In New Zealand, Māori have the highest rates of chronic pain compared with other population groups, and chronic pain is more prevalent in areas of high socio-economic deprivation (48, 49). Acute and chronic non-cancer pain rates in Australia and New Zealand are likely to continue to rise, related to the ageing population, lifestyle changes leading to obesity and inactivity, and the epidemiological shift from infectious diseases to non-communicable diseases (such as diabetes and arthritis) (43, 50–52). Advances in treatment of cancer have led to an increase of painful neuropathic conditions (53).

Medical practitioners need to recognise at-risk populations, and implement effective strategies for acute and chronic pain assessment and management so as to reduce the public health burden of pain (54).

### 2.2. Responsibility for developing and articulating pain medicine learning outcomes

#### 2.2.1. The influence of the professional regulatory system on the inclusion of pain medicine in the medical curriculum

Professional accreditation bodies significantly influence curriculum design through the regulations and standards that they set (55). Accreditation is the process whereby organisations set standards to ensure that graduates are competent and safe to practice (56). The medical curriculum must meet the demands of the accrediting and professional bodies with respect to defined graduate outcomes. Influencing professional bodies to incorporate pain medicine competencies in entry-to-practice registration and maintenance of certification is likely to have a major impact on pain education and clinical practice (55, 57). It appears that regulatory bodies in Australia and New Zealand have not directed curricular requirements to integrate pain medicine into the curriculum. The Australian Medical Council (AMC) is responsible for developing standards, policies and procedures for the accreditation of medical programmes for Australia, and sets a framework around which medical education providers structure their individual programmes (58). The New Zealand Medical Council (NZMC) monitors the training of medical students in New Zealand (59). However, neither the AMC nor the NZMC has specifically defined in detail the outcomes that a student must demonstrate for graduation (58, 59). A defined pain medicine curriculum is therefore not a mandatory part of medical degrees in Australia and New Zealand.

Similarly, competencies in pain medicine have not been prioritised by regulatory bodies in Australia and New Zealand. Medical Deans Australia and New Zealand Inc, the eminent body representing entry-level medical education in Australia and New Zealand, endeavours to bring together stakeholders from all levels of medical education and training to prioritise future medical workforce planning (30, 60). In 2020, the Medical Deans' Medical Education Collaborative Committee identified a set of core competencies describing the foundational skills and knowledge required for final-year medical students to be ready for internship (61). No specific pain management core skills were identified apart from “prescribing analgesic medication (opioid and non-opioid)”. The report specified that students should be able to demonstrate the knowledge of safe prescribing of high-risk medicines such as analgesics in a simulated experience or environment (such as an objective structured clinical examination), and at the time of graduation, be able to perform this competency under indirect supervision (61).

Likewise, entry-to-practice competencies that specifically identify pain-related knowledge, skills or attitudes are minimal or mostly absent in regulatory requirements for medical graduates in the United States of America (USA), Canada and the United Kingdom (UK) (57, 62). This is one of the major reasons that comprehensive pain management content is not mandatory in the medical curriculum in these countries (12–14, 63). Entry-to-practice competency requirements related to health science undergraduate training in Canada were examined in 2013 (55). While dentistry and nursing students were required to complete a number of pain-specific competencies, no regulatory requirements related to pain were found for medical students (55).

Core competencies for pain management have been accepted across a number of health professions and speciality professional organisations (such as the International Association for the Study of Pain [IASP], American Academy of Pain Medicine, American Society for Pain Management Nursing, American Council of Academic Physical Therapy, Royal College of Nursing and UK Physiotherapy Pain Association) (57, 64). Systematic change is likely to follow in terms of integration of pain education into the curriculum when accrediting bodies prioritise the need for medical students to display competencies in pain management (57).

#### 2.2.2. Legal, ethical, and social issues related to pain medicine education

The consequences of not treating chronic pain can be severe, leading to significant deterioration in health-related quality of life and psychological wellbeing (65–68). The social consequences of persistent pain include breakdown of family and marital relationships, altered social role and social isolation (69, 70).

The economic cost of persistent pain on society is enormous. The total cost of chronic pain in 2018 in Australia was estimated at AUD$139.3 billion and 7% of total health system expenditure (cardiovascular disease accounted for 10% in a similar period), and up to $15 billion in New Zealand in 2016 (43, 71, 72). This cost included loss of productivity at work, burden of disease costs and healthcare costs, as well as welfare benefits and loss of taxation revenue (51, 71). Economic costs are attributable to the significant adverse effect on people who experience pain, but also on those caring for them, as well as friends and family, co-workers, employers, charities and governments. Pain negatively affects work productivity for both the patient and the carer. Loss of productive time can be explained by reduced performance at work, as well as by absence from work and premature retirement (73, 74).

There are also risks of harm associated with inappropriate treatment of chronic pain. While the value in using opioids for acute and cancer pain is accepted, opioids are increasingly being prescribed for chronic non-cancer pain despite an absence of evidence regarding the long-term efficacy or effectiveness (75). There are significant harms associated with the long-term use of opioids such as physical dependence, addiction, opioid-induced hyperalgesia and overdose (unintentional or intentional) (76). Medical practitioners face legal scrutiny in terms of opioid prescription, including over- or inappropriate prescription (77). There has been a substantial increase in prescription of opioid medications for chronic non-cancer pain in Australia and New Zealand in the past 20 years, with a parallel increase in opioid abuse, addiction and overdose deaths (76, 78, 79). Internationally, substantial practice and knowledge gaps of prescribing physicians have been identified, such as prescription of transdermal fentanyl in opioid-naive patients, or failure to discontinue opioids if ineffective for relieving pain (80). An inquest into the death of a patient in South Australia in 2015 found that the death was preventable and occurred as a result of prescribed opioid toxicity (81). Medical practitioners have recently been reprimanded in Australia over the inappropriate used of opioids and ketamine (an anaesthetic agent) (82–84).

Medical schools have a legal and ethical duty to teach pain management in a comprehensive manner in order to equip graduates with technical, cognitive, emotional and reflective skills to adequately manage people with pain needs (85).

#### 2.2.3. Governmental support for developing pain medicine education

Pain has a low medico-political profile worldwide (9, 50, 68, 86). At present, the provision of pain care in Australia has been described as fragmented; in particular, chronic pain care is lacking a coordinated approach (87). Some changes are taking place in Australia, including the 2018 National Strategic Action Plan for Pain Management, supported by the Australian Government, which provides support for improved pain medicine education at medical schools in Australia (5). The Action Plan was developed by over 25 organisations, including those related to pain medicine, allied health, drug and addiction medicine, mental health, rural health, general practice and pharmacy as well as consumers and carers and is supported by the Australian Government (5). A key goal of the Action Plan (2018–2021) was to ensure that health practitioners are well-informed on the best practice evidence-based pain management and supported to deliver this care (5). The Action Plan aimed to achieve this goal by developing an overarching education strategy to promote evidence-based pain management education across health practitioner disciplines (5). This included standardisation of teaching curricula at universities and a focus on value-based health care (88).

No comprehensive population health-level strategy currently exists in New Zealand to tackle the magnitude of the problem of pain with coordinated strategies for pain prevention, treatment, education, reimbursement and research (43). It is critical that government agencies prioritise a coordinated national strategy and provide financial support for pain education to address the unnecessary burden of unrelieved pain (89, 90).

### 2.3. Expectations of pain medicine competencies in the workplace

What challenges are medical graduates likely to face when providing pain treatments in their local health system? There is no clinical specialty where the basics of pain management are not relevant because acute and chronic pain are features of each of these disciplines (7).

#### 2.3.1. Hospital setting

It has been established that pain is common and often undertreated in both medical and surgical hospital inpatients in Australia (91–94). International studies report that acute pain is the main complaint of patients seeking treatment at an emergency department of a hospital, with approximately seven out of 10 patients attending because of severe pain (50, 95, 96). A prospective observational study of patients in Australia found that 47% of patients continued to experience moderate to severe pain one week after surgery (97). A further study in Australia showed that severe acute pain was reported by 56% of patients up to three days after orthopaedic surgery (98). Corresponding figures for acute pain prevalence in New Zealand have not been published.

Newly graduated medical practitioners in Australia and New Zealand (hereafter referred to as interns), are directly responsible for managing patients with pain presenting to hospital (99–101). A mixed methods study to better understand the clinical placement experience of prevocational doctors in Australia found that interns prescribed pain therapies and participated in discharge planning for most of their patients (100). For some of their patients, they implemented a management plan and prescribed the patients' medication throughout their stay (100). A survey of new interns in Australia found they frequently performed pain management tasks without direct supervision during the first year after graduation (99). A further study in Australia identified that levels of supervision decreased during night and weekend shifts and were dependent on service demands (102). In a survey undertaken in New South Wales, Australia, 70% of interns stated that they would be expected to initiate preliminary investigation, management or treatment for post-operative pain without supervision (103). Inadequate monitoring of interns' prescribing of analgesics has been described both in New Zealand and internationally (101, 104).

A study of opioid prescribing at a hospital in Australia showed that patients received inadequate analgesia because of medical practitioners' limited knowledge of pain assessment, opioid dose titration, available opioid preparations, lack of experience of multimodal analgesia and attitudes to opioids and pain relief (91). Patients are at risk of harm when interns lack of knowledge regarding analgesic medications. A study of junior doctors' opioid prescribing practices in New Zealand that found dose errors were common (54%) with 19% of these likely harmful and 4% potentially lethal (101). This appears to be a widespread problem among junior doctors internationally (105). In Australia, discharge prescribing is often delegated to junior doctors, and high doses of opioids in excess of patient are routinely prescribed (106).

There are similar reports of new graduates from medical schools in the USA with varying degrees of readiness to provide adequate pain management for their patients (107). Interns in the USA are generally poorly prepared to evaluate and treat acute pain, and find the complex problem of acute-on-chronic pain overwhelming (107). A survey of interns in the USA found that 78% reported a lack of training and competency in the prescription of opioids for chronic non-cancer pain (108).

#### 2.3.2. Primary care

Specialist pain medicine resources are limited in Australia and New Zealand (43, 44, 109). It is therefore essential that patients with pain receive timely and appropriate care by non-pain specialists in the primary care setting (7).

Internationally, general practitioners have reported inadequate training regarding pain management, and have expressed difficulties with assessing and managing chronic pain, especially for their elderly patients and those requiring opioid treatment (110–113). Many patients feel that healthcare practitioners lack relevant knowledge regarding chronic pain and are dismissive of their individual pain needs (8, 114).

### 2.4. Need for collaboration

Proposals have been made both nationally and internationally to mobilise medical education stakeholders (patients, medical practitioners, allied health professionals and governmental bodies) to integrate a formal comprehensive pain medicine curriculum into medical school training (5, 33, 50, 62, 87, 115). An academic–clinical partnership is needed to develop effective collaborative approaches to improving pain medicine competencies of medical students.

An Australian study examining beliefs and clinical practice behaviours related to low back pain among multidisciplinary health professional students recommended more consistent alignment of evidence-based education regarding low back pain across disciplines (94). The problem of limited integration of pain content in pre-licensure health sciences curricula such as nursing, dentistry, occupational therapy, physiotherapy, pharmacy and social work has been identified in Canada, Europe and the USA (73, 81, 82, 91–93). The delivery of effective pain management can be complex and requires multidisciplinary team approaches (116). It is important that health professional students are provided with a common understanding of the basic principles of pain management in order to prepare them to work as part of an integrated multidisciplinary team (8, 117).

On a national scale, the Faculty of Pain Medicine of the Australian and New Zealand College of Anaesthetists (FPM ANZCA) has partnered with the Australian Government (through the Therapeutic Goods Administration) to support pain education for nurses and medical students. The Better Pain Prescribing initiative involves funding for nurses and medical students to access the Better Pain Management e-learning programme on the multidisciplinary, patient-centred approach to the assessment, diagnosis and management of people experiencing pain (88, 118, 119).

Medical schools need to collaborate with different stakeholders (academics, medical training regulators, professional medical colleges, and patient/consumer groups) to meet their responsibility for ensuring that pain medicine education is effectively integrated into the medical curriculum.

## 3. Dimension 2: defining and understanding pain medicine capabilities

The second dimension involves identifying sets of learning outcomes to specify the pain medicine knowledge, capabilities and attributes needed by health professionals to competently participate in high-quality, relevant and comprehensive health systems. The curriculum needs to connect knowledge with experience and practice to be responsive to the changing needs of the increasingly complex health system yet adaptable to patients with pain in the local context.

### 3.1. Currently available pain curricula

The original IASP curriculum was updated and entered its fourth edition in 2017 (120, 121). This IASP Curriculum Outline on Pain for Medicine is intended as a guideline for medical school curriculum planning, to draw attention to key pain concepts that should be taught during the medical training (121).

According to IASP, principles that should guide the pain curriculum for the entry-level physician are:

• Pain is multidimensional requiring comprehensive and ongoing assessment and effective management.
• Physicians play an essential role in the prevention, diagnosis and management of acute and persistent pain (121).

The specific objectives of this entry-level pain curriculum are:

(1) Recognize pain medicine as a necessary field in clinical practice for acute and persistent (chronic) pain conditions
(2) Understand the basic science of pain-processing components such as anatomy, physiology, and pharmacology
(3) Identify clinical presentation of acute and persistent pain syndromes or conditions
(4) Recognize the multidimensional aspects of the pain experience and its related management
(5) Understand pain management options appropriate for individual patients according to medical condition, medicine availability, risk-benefit balance, cost-effectiveness, culture, mental status, and evidence of efficacy
(6) Know the indications, contraindications, and risks of the primary elements of multimodal pain management
(7) Learn effective interaction with multi-professional teams involved in practicing pain medicine
(8) Practice pain medicine according to ethical principles (121).

The IASP Curriculum Outline on Pain for Medicine complements the European Pain Federation (EFIC) Pain Management Core Curriculum for Medical Students, which gives a more detailed breakdown of educational objectives, structure, content, number of teaching sessions and suggestions for delivery (122).

**Figure 2:**
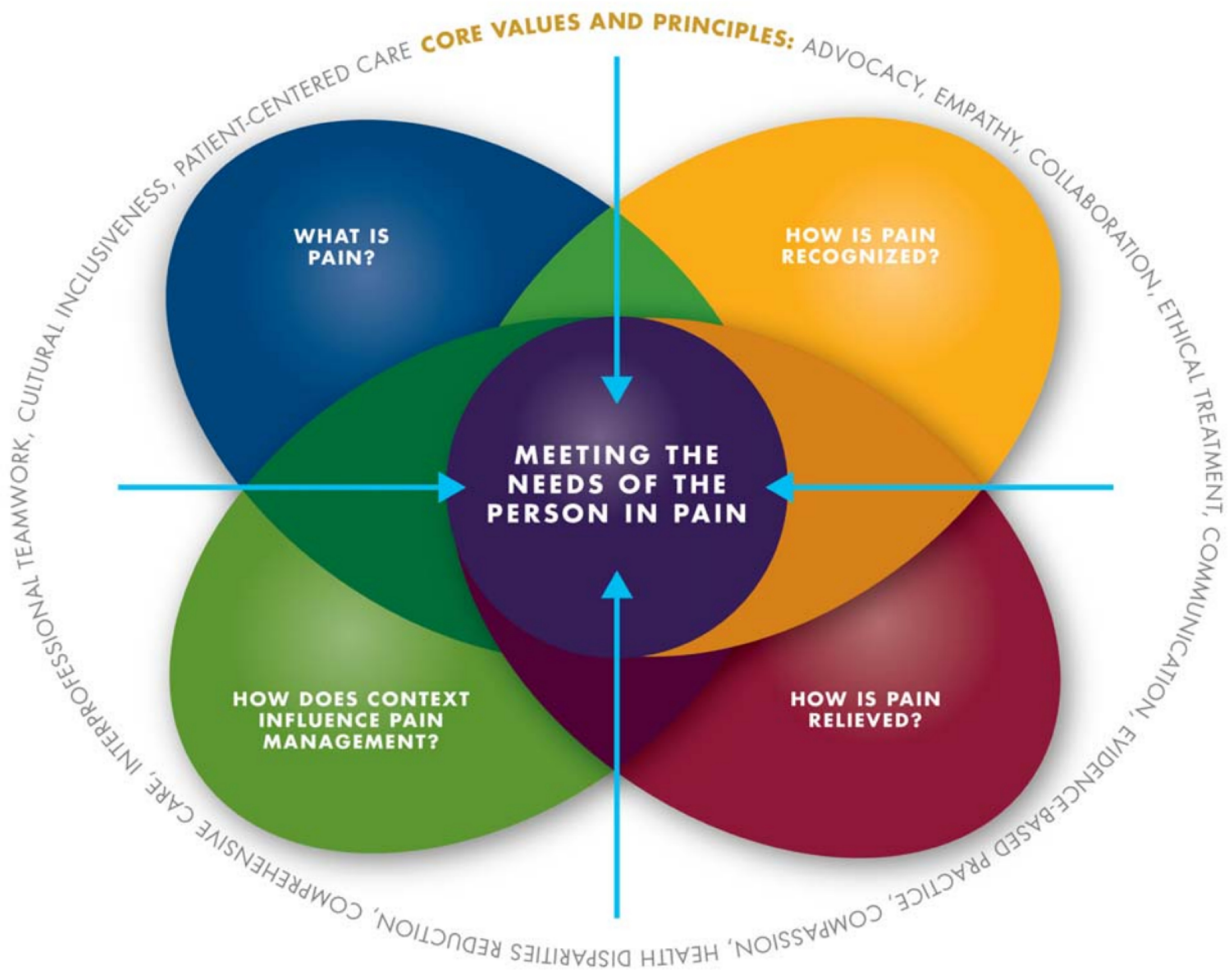
The core competencies for pain management (116). The core competencies are categorised within four domains. Core values and principles are embedded into all domains and competencies. Figure prepared by Ian Koebner, PhD, MS, and used with permission of Professor Scott Fishman, MD, Principal Investigator of the Expert Interprofessional Pain Competencies Consensus Group.

With the advances in the educational research and emphasis on competency-based education, pain management experts and educators became aware of the absence of pain management core competencies for entry-level health professional students (2, 123). It was felt that this deficiency was possibly one of the reasons for the lack of pain education in training programmes (2). In 2012, the Expert Interprofessional Pain Competencies Consensus Group (EIPCCG) comprising leaders from multiple professions with expertise in pain management, education science and development of evidence-based consensus came together to develop core competencies in pain assessment and management for entry-level health professional education (2). The recommended pain management competencies were categorised into four domains: multidimensional nature of pain, pain assessment and measurement, management of pain, and context of pain management (see Figure 2) (2). These domains address the fundamental concepts and complexity of pain; how pain is observed and assessed; collaborative approaches to treatment options; and application of competencies across the life span in the context of various settings, populations and care team models (2). These core competencies were based on the IASP interprofessional core curriculum (124).

### 3.2. Integrating pain medicine core competencies into medical curricula

The EFIC and IASP core curriculum have been recommended by expert pain researchers as a suitable structure for pain teaching in the undergraduate curriculum (14, 17, 18). The IASP Curriculum Outline on Pain for Medicine has been used as a reference to develop content of pain management courses in medical schools in Greece, the USA (Johns Hopkins University, Virginia Commonwealth University, New York University, University of Washington, State of Michigan medical schools), Finland, the UK and Canada (University of Toronto) (16–21, 23, 125–127).

In 2016, the EIPCCG pain management core competencies formed the basis of the document Strengthening Pain Content in Medical School Curricula, which was developed by an expert panel as a tool for integrating pain management content specifically into medical school curricula (128). Potential teaching methods and suggestions for education strategies and content were identified for each learning goal (128). The document also mapped the pain management core competencies with the Association of American Medical Colleges' Physician Competency Reference Set (128). The EIPCCG pain management core competencies have also been used as a framework for postgraduate continuing professional development for pain educators and clinicians (129).

A workgroup from the University of California was tasked to develop a set of core educational competencies to address pain, substance overuse disorder and safer opioid prescribing for adoption across the six medical schools in the academic health system (130). The final set of University of California pain and substance use disorder competencies was compiled in 2019 (130). Both medication management and nonpharmacological strategies to address pain and substance overuse disorder were included.

A curriculum audit of pain medicine education at medical schools in Australia and New Zealand showed that while 42% of medical schools had partially implemented the recommended IASP Curriculum Outline on Pain for Medicine, none had successfully achieved full integration of this comprehensive curriculum (121). Pain medicine curricula in Australia and New Zealand focused mainly on the neurophysiology, clinical assessment and biomedical treatment of pain, primarily using analgesics (131). A focused review of pain medicine education at medical schools internationally noted similar gaps in the breadth of core topics between the IASP-recommended pain medicine curricula and documented educational content (11–13, 15, 16, 131). These international surveys found that essential topics reflecting the biopsychosocial framework and multidisciplinary treatment of pain were underrepresented at most medical schools (132).

Medical students in Australia and New Zealand display gaps in proficiency in pain medicine knowledge, skills and attitudes, especially with regard to clinical pharmacology, understanding evidence-based pain management options for individual patients and concepts such as allodynia and central sensitisation (10, 133, 134). International studies have shown a similar lack of pain medicine competencies of medical students (135–138).

Pain medicine education needs to ensure that medical graduates are confident in their ability to respond to patients with pain, understand how the patient is experiencing pain, and recognise their own cultural and emotional response to pain (23, 139).

## 4. Dimension 3: teaching, learning and assessment

The third dimension of the curriculum framework considers the development of appropriate learning, teaching and assessment strategies that are necessary to ensure that medical practitioners of the future develop the required knowledge, skills and attitudes to treat the diverse needs of patients' experiencing pain.

### 4.1. The learning and teaching process

Pain management is complex and requires an understanding of the multidimensional aspects of the pain experience and its related management (121). Clearly defined objectives are important to connect learning activities and content with the pain medicine competencies doctors will require in clinical practice (29, 34, 132).

Traditional teaching methods such as lectures and seminars are commonly used for teaching the foundational concepts of pain management (basic sciences of pain processing and pharmacological therapy) to provide a well-structured base on which further knowledge is built (140, 141). More sophisticated strategies are likely to be required to provide opportunities for students to learn advanced competencies such as delivery of patient-centred pain management within the multiprofessional teams, empowerment of patients to self-manage their pain, explaining concepts such as central sensitisation to patients, and adapting pain assessment and management to the unique needs of special populations (2, 141). Formative OSCEs and structured clinical instruction modules have been used to improve pain medicine competencies of medical students (20, 142–146). Individual medical schools in the USA and Canada have developed dedicated pain modules using small-group discussions, expert-led sessions and patient interactions to improve students' clinical skills, attitudes and knowledge with regard to pain assessment and management (20, 23, 125). Case-based teaching and problem-based learning have also been used to develop the pain management skills necessary to apply knowledge in clinical situations (19–21, 125, 144, 145, 147–150). Expert pain medicine educators have stated that students are more likely to be engaged in pain education with student-centred learning and problem-based learning that includes the use of personal stories of pain (151).

The use of high- and low-fidelity simulation to provide students with a variety of real-life situational experiences (for example managing pain crises or challenging patient scenarios), and exposure to group interdisciplinary pain management planning can improve levels of critical thinking ability (125, 141, 152–156).

Pain provokes a strong negative response primarily on the person experiencing it directly, but can also impact primary caregivers and medical students (144, 157–160). Medical schools need to provide opportunities for students that will encourage positive emotional development and resilience relevant to pain care in conjunction with clinical pain medicine knowledge (161). Effective pain management requires medical practitioners demonstrate empathy, foster productive communication and nurture positive relationships (162). Role playing, motivational interviewing training, communication skills training and improved observational skills training are educational tools that have been recommended to help build empathy (163). Teaching methods such as writing a brief pain narrative, describing pain depicted in a fine-art image, and assessing personal responses to the experience of pain have improved students' awareness of the affective dimensions of pain while fostering their emotional development (161). Journaling, discussion groups and structured reflection have also been used by an individual medical school with positive outcomes on pain competencies (161).

Exposing students to different clinical learning opportunities, such as multidisciplinary pain clinics, general practice clinics, hospital and home visits, helps students understand pain management in the context of varied patient populations, settings and care teams (2, 18, 29, 141, 142). This exposure is important so that students see the continuum of pain care and the impact of pain on patients outside the hospital setting (29, 142).

However, careful selection of clinical placement is important as there is the potential for medical students to be taught by clinicians have not been adequately trained in pain medicine, and therefore providing suboptimal pain treatment and demonstrating negative attitudes towards their patients in pain (8, 164, 165). The challenge is to ensure that medical students are exposed to meaningful clinical learning opportunities in pain medicine.

Web-based pain medicine resources are being developed (19, 142, 147, 148, 166). These modules have been useful for improving medical students' pain competencies in acute, cancer, paediatric, chronic non-cancer and chronic low back pain. The e-learning resources were recommended because they provided resources to simulate authentic real-world contexts and had the potential to facilitate learning face to face or in remote settings (166). Increased use and sharing of online pain medicine education resources could potentially address the staff and learning resource deficit that has been identified (167–170). These e-resources need to be cost-effective and updated regularly.

There is no gold standard for delivering pain medicine education and each medical school would need to determine which model is most suitable for their local context. Pain education could be sequenced from more foundational concepts at the beginning of the medical course to more advanced curricula towards the final years of the course, with required competencies attained at different stages. “Flipping the pain curriculum” has been suggested, so instead of the standard approach of beginning with and emphasising pathophysiological pain processes, students would be initially exposed to the epidemiology of pain and disability, as well as the social and psychological aspects of pain in society, and then move to the more detailed biomedical aspects of pain management (171).

A flexible modular approach integrated over the entire medical curriculum may be the best way to structure the pain curricula for some universities, with pain medicine a common theme throughout the curriculum and different specialities plus a dedicated pain medicine rotation (12, 18, 154). Pain medicine education needs to be systematically integrated into all disciplines since pain is ubiquitous in clinical settings. A curriculum map might be useful to sequence pain curricula and improve cohesion of the pain medicine teaching throughout the medical training programme (172).

### 4.2. Assessment

Pain medicine competencies need to be assessed for formative and summative purposes to encourage learning, to enhance the importance of pain medicine education, to identify education gaps in the curriculum with respect to pain medicine and to ensure that new graduates are competent and safe to enter the workforce (173, 174).

The Pain Medicine Assessment Framework (PMAF) has been recommended to encourage a systematic approach to the task of assessing medical students' pain medicine competencies (121, 175). This framework emphasises the core pain medicine competencies recommended for pre-licensure health professionals by the EIPCCG (2) as well as the IASP Curriculum Outline on Pain for Medicine (121).

Assessments need to focus not only on pain medicine knowledge but also on clinical skills and attitudes. Written assessments such as multiple choice questions are reliable and practical to assess cognitive pain medicine knowledge and higher order thinking (such as applying knowledge to clinical situations) (173, 176–178). The OSCE assessment has been used effectively to assess medical students' pain competencies such as clinical knowledge, communication, empathy and attitude in a variety of contexts (acute, low back and cancer pain) (20, 145, 147, 179, 180). Progressive medical schools have developed alternative assessment methods (such as reflective journals, vignettes and portfolios) and multifaceted assessment processes to measure multiple domains of competence in clinical pain medicine practice (23, 125, 148, 173).

Internationally, pain medicine learning is likely to be assessed using written examinations, if undertaken at all (11, 173). OSCEs and practical assessments for pain medicine are used by very few medical schools internationally; and by less than 10% of medical schools in Europe (14, 16, 131, 181). There is no national licensing examination in Australian and New Zealand, so medical schools need to develop their own assessment processes to ensure that graduates possess the range of pain medicine competencies to meet the complex needs of people in pain (30, 131).

### 4.3. Interprofessional education (IPE)

Pain assessment and management provide an excellent model of interprofessional teaching and learning because of the multidimensional nature of pain (182). IPE is not fully integrated into the medical curriculum in Australia and New Zealand, and often exists as diverse discreet standalone programmes at individual universities (27, 183–186).

IPE has been shown to be effective for improving medical students’ pain competencies in a variety of settings, including general pain management, paediatric pain and acute pain (125, 146, 187, 188). The interfaculty pain curriculum at the University of Toronto, Canada, includes interprofessional small-group sessions focused on developing assessment skills and management plans for patients using standardised patients (21). The e-learning Pain Education Interprofessional Resource also delivered at the University of Toronto has been shown to improve health professional students' pain knowledge and understanding of collaborative care (166). Medical schools need to build interprofessional teaching and learning opportunities into the medical curriculum to reinforce the importance of health professionals working together to effectively manage pain (187).

## 5. Dimension 4: supporting institutional delivery of pain medicine education

The last dimension of the curriculum framework is concerned with the influence of local university context on pain medicine education including the diverse strategic vision of medical schools, access to pain medicine resources and clinical teaching opportunities, and research funding. This involves the historical, political, social and organisational values of the educational institution, which will have a significant impact on curriculum design (34).

### 5.1. Value systems of individual education institutions: prioritising pain medicine

Pain medicine is a relatively new healthcare field, but is rapidly evolving (63). In particular, the understanding of transition from acute to chronic pain, and translation of promising scientific advances into effective diagnostic, preventative and therapeutic strategies for patients have dramatically improved in the past three decades (189, 190). Identification of peripheral and central nociceptive processes, discovery of endogenous neurochemicals and recognition of the role of the immune system in the maintenance of pain have furthered the understanding of pain mechanisms, diagnosis and treatment (132). Internationally, there is a continuing gap between what is known about pain medicine and the translation of this into clinical practice (62, 190). In general, despite robust evidence for a biopsychosocial model of pain, many medical practitioners continue to focus on a purely biomedical approach to pain (191). Pain is often seen as a symptom of a disease and therefore given a low priority by medical practitioners (9). Pain medicine education needs to be prioritised by medical schools to ensure that future medical practitioners are able to effectively and safely manage pain. This will require concerted collaborative effort and advocacy to ensure that greater time and resources are allocated to pain teaching.

Raising the value of pain medicine education necessitates increased profile of pain medicine in the curriculum and the recognition of pain medicine as an independent discipline rather than the domain of subspecialty training. The discipline of pain medicine was recognised in Australia as a medical specialty in its own right in 2005, and was accredited as a scope of practice in New Zealand in 2012 (192). Currently, the University of Notre Dame Australia has the Churack Chair of Chronic Pain Education and Research, and the University of Sydney has a chair in pain medicine. There is no chair in pain medicine in New Zealand.

To support the development of pain medicine curricula at medical schools in Australia and New Zealand, the FPM ANZCA offers an annual prize to the best medical student in pain medicine at each medical school. Most medical schools do not make use of this opportunity to raise the profile of pain medicine (193).

Each medical school has a set of norms and values that underpin its curriculum (194). Explicit values can be apparent in the formal curricula, such as course content, hours, requirements and evaluation. In Australia and New Zealand, there appears to be a lack of dedicated pain medicine modules, minimal learning time and little evaluation of pain medicine competencies as a requirement for graduation (131). The medical curriculum has been described as overcrowded with multiple competing priorities, so it may be difficult to find space for a new pain medicine programme in an already compacted course (195, 196).

Important learning also occurs *via* opportunistic teaching during clinical ward rounds (the informal curricula). Students learn by example from interactions with their teachers, also termed the hidden curriculum. This hidden curriculum pertains to what is tacitly acquired by example during training as opposed to the formally explicit teaching that the medical school intends to deliver (197). Lack of teaching or clinical exposure on a topic also portrays a value judgement (null curricula) (29). The null curriculum of pain medicine would be the absence of teaching regarding the management and assessment of patients experiencing pain from the formal curriculum (29). The imbalance of topics at medical schools has been attributed to a failure to recognise the prevalence of patients' experiencing pain in most primary care practices and indeed in most specialities (18).

Students continue to learn from senior medical practitioners who have not been adequately trained in evidence-based pain management (9). For example, medical professionals consistently tend to underestimate pain and the suffering of their patients, and this tendency is more pronounced when the patient reports severe pain and depression (198, 199). This has affected medical students' capacity to trust their patients' accounts of their pain (165). The hidden curriculum has been mentioned in the context of medical school pain education, where students stated that pain was viewed as a nuisance rather than an important symptom and disease in its own right (164). Medical students have also described a hidden curriculum that suggests that chronic pain patients lack educational value and are too difficult to treat (165). Students indicated that since their training primarily emphasised objective measurements, diagnosis and curative treatment, they were unprepared to deal with the “subjectivity” of pain and inability to cure chronic pain (165).

Culture is a powerful force in shaping beliefs and behaviours about pain (200). In New Zealand, one model for understanding Māori health is the concept of te whare tapa whā – the four cornerstones (or sides) of Hauora Māori. In a traditional Māori approach, the inclusion of wairua (the spiritual dimension), the role of the whānau (family) and the balance of hinengaro (mind) are as important as the physical manifestations of illness (taha tinana).

Medical students need to be made aware of their own biases and prejudices towards patients with pain (164). For instance, recent research in New Zealand has highlighted that Māori adults who experience chronic pain are not being offered holistic explanations about the causes of their pain, and are instead being prescribed analgesics at the expense of best practice treatments (201). It is imperative that medical schools address cross-cultural pain education to ensure issues such as conflicting perceptions regarding pain expression and disparities (in assessment, analgesic requirements and treatment) (200).

### 5.2. Staff resources

The literature supports the concept that pain medicine education is best provided by specialists (medical and allied) trained in pain medicine (62, 202). Lack of qualifications of teaching staff at medical schools to provide pain medicine education has been highlighted internationally (62, 203). There is a lack of qualified SPMPs in Australia and New Zealand, particularly in the rural setting (43, 44). Medical schools in Australia and New Zealand spread students over a number of training centres, including rural sites (204). A lack of allied health professionals and general practitioners with professional training in pain management in rural districts has also been identified (205, 206). For students to effectively work in partnership with other health professionals when treating people with complex pain presentations, they need to understand and value other health professionals' roles and expertise (116).

Medical schools need to commit to building a team of medical and allied health pain specialists who are equipped with the skills and teaching resources required to deliver comprehensive pain medicine curricula. It is also clear that continuing professional development for medical practitioners who oversee clinical learning opportunities would be useful to ensure that medical students are provided with consistent evidence-based pain medicine teaching throughout their medical training.

### 5.3. Locally adapted learning and teaching resources

Changes to the curriculum require much planning and financial investment, which may be prohibitive because of a lack of resources. Calls by specialist colleges (such as the FPM ANZCA) for changes to the curriculum to include more pain content may be ignored due to a lack of support and resources offered by these bodies (87, 154). The need for more research and development of pain education resources has been identified previously (89, 207). A systematic review of online pain resources for health professionals found that those available were helpful in improving learner knowledge and skills (207), however more support is needed for the development and distribution of pain medicine teaching resources to medical schools across Australia and New Zealand.

Curriculum designers will need to adapt these pain curricula to suit the needs of the local community they serve. For instance, in New Zealand, Māori have a pedagogical concept of “Ako” that acknowledges the way that new knowledge grows out of shared learning experiences; recognizing the knowledge that both teachers and learners bring to the learning environment (208). It affirms the value of building caring and inclusive learning communities. Studies have shown the need for culturally responsive pain management resources for people with persistent pain in New Zealand (201, 209). The pain medicine curriculum would need to be flexible in design for it to be incorporated into the diverse landscape of medical education in Australia and New Zealand.

### 5.4. Local networking

Pain specialists in the UK have advocated for local clinical and educational champions for pain education to build strong alliances with deans of medical schools and non-specialists in pain in their local schools to facilitate the incorporation of pain education into the curricula (7, 210). Medical schools would benefit from identifying a local pain champion to drive integration of pain medicine education into the medical curriculum.

## 6. Conclusion

Change is needed in the way pain medicine is taught at medical schools across Australia and New Zealand. It is crucial that a more formalised approach to the development and delivery of a comprehensive pain medicine curriculum is provided during pre-licensure training to ensure that graduates are adequately prepared for their future workplace responsibilities.

The Pain Medicine Curriculum Framework for improving pain medicine education presented in this paper will assist curriculum designers in Australia and New Zealand, and internationally, in the ongoing process of ensuring that medical graduates meet the professional and ethical challenges that arise in caring for those in pain.
